# Manual Ventilation and Sustained Lung Inflation in an Experimental Model: Influence of Equipment Type and Operator’s Training

**DOI:** 10.1371/journal.pone.0148475

**Published:** 2016-02-09

**Authors:** Cristiane do Prado, Ruth Guinsburg, Maria Fernanda Branco de Almeida, Renata Suman Mascaretti, Luciana Assis Vale, Luciana Branco Haddad, Celso Moura Rebello

**Affiliations:** 1 Department of Pediatrics, School of Medicine, University of Sao Paulo, Sao Paulo, Sao Paulo, Brazil; 2 Brazilian Society of Pediatrics, Neonatal Resuscitation Program, Sao Paulo, Sao Paulo, Brazil; 3 Department of Pediatrics, Hospital da Vila Santa Catarina, Sao Paulo, Sao Paulo, Brazil; 4 Department of Pediatrics, Hospital Israelita Albert Einstein, Sao Paulo, Sao Paulo, Brazil; 5 Department of Pediatrics, Paulista School of Medicine, Federal University of Sao Paulo, Sao Paulo, Sao Paulo, Brazil; Azienda Ospedaliero-Universitaria Careggi, ITALY

## Abstract

**Aim:**

To compare the influence of devices for manual ventilation and individual experience on the applied respiratory mechanics and sustained lung inflation.

**Methods:**

A total of 114 instructors and non-instructors from the Neonatal Resuscitation Program of the Brazilian Society of Pediatrics participated in this study. Participants ventilated an intubated manikin. To evaluate respiratory mechanics and sustained lung inflation parameters, a direct comparison was made between the self-inflating bag and the T-shaped resuscitator (T-piece), followed by an analysis of the effectiveness of the equipment according to the participants’ education and training.

**Results:**

A difference between equipment types was observed for the tidal volume, with a median (interquartile range) of 28.5 mL (12.6) for the self-inflating bag and 20.1 mL (8.4) for the T-piece in the instructor group and 31.6 mL (14) for the self-inflating bag and 22.3 mL (8.8) for the T-piece in the non-instructor group. Higher inspiratory time values were observed with the T-piece in both groups of professionals, with no significant difference between them. The operator’s ability to maintain the target pressure over the 10 seconds of sustained lung inflation was evaluated using the area under the pressure-time curve and was 1.7-fold higher with the use of the T-piece. Inspiratory pressure and mean airway pressure applied during sustained lung inflation were greater with the self-inflating bag, as evaluated between the beginning and the end of the procedure.

**Conclusion:**

The T-piece resulted in lower tidal volume and higher inspiratory time values, irrespective of the operator’s experience, and increased the ease of performing the sustained lung inflation maneuver, as demonstrated by the maintenance of target pressure for the desired period and a higher mean airway pressure than that obtained using the self-inflating bag.

## Introduction

Approximately 136 million babies are born worldwide each year, and between 3% and 6% of them have difficulty establishing and maintaining effective breathing [1] and require mechanical ventilation in the delivery room [2]. In fact, it is estimated that approximately 20% of premature infants receive positive pressure ventilation shortly after birth [3,4].

The premature infant's lung is vulnerable to injury from the use of excessively high tidal volumes and inspiratory pressures, particularly in the absence of positive end-expiratory pressure (PEEP), even when applied over just a few respiratory cycles [5–7] This intervention can contribute to the onset of an inflammatory cascade and can result in lung injury [8,9]. Therefore, proper adjustment of these parameters during the resuscitation process is critical to minimize the possibility of complications.

The application of positive pressure for a longer time to obtain uniform lung aeration and adequate gas exchange may be beneficial due to the high resistance to the displacement of lung fluid into the distal airways during the first breath [10]. With this possibility in mind, the concept of sustained lung inflation (SLI) in neonatal resuscitation was developed and involves the application of a constant pressure to the airways, between 20–25 cmH_2_O above PEEP, for a period of 10 to 20 seconds [4]. Although some authors consider SLI controversial [11], there is evidence to suggest that in the absence of SLI, initial aeration is gradual and limited to small areas of the lungs, making these areas susceptible to injury if high pressures and short inspiratory times (Ti) are used [12]. Moreover, the use of SLI associated with PEEP may decrease the need for intubation within the first 72 h of life [13], the use of surfactant and postnatal corticosteroids and the duration of mechanical ventilation [1].

The equipment used for manual ventilation includes the self-inflating bag (SIB), flow-inflating bag and T-shaped resuscitator (T-piece). However, the evidence regarding which of these options is the best choice is limited [1]. The SIB is the most commonly used in neonatal resuscitation, although the T-piece is more accurate in maintaining peak inspiratory pressure (PIP) and PEEP during manual ventilation and the application of SLI [14–16]. Furthermore, there is evidence to suggest that the use of the T-piece decreases the intubation rate and the PIP applied [17].

Although it is well established that PIP and tidal volume (Vt) values above the recommended limits represent key risk factors for lung injury [5,9,8], only a few studies to date have investigated how respiratory mechanic variables differ among equipment types [18]. Furthermore, few studies have quantified the relative capacity of the T-piece to maintain respiratory parameters at constant levels.

The objective of this study was to evaluate the magnitude of the differences observed between the SIB and T-piece in respiratory mechanics during manual ventilation and the SLI maneuver. We also present a new approach for comparing the capacity of these types of equipment to maintain SLI for 10 seconds, which provides a method to quantify the real differences in efficacy between the two devices. Finally, we also evaluated the effect of instructor education and training in neonatal resuscitation on the effectiveness of manual ventilation and the ability to perform SLI using both types of equipment.

## Materials and Methods

### Design and study population

This experimental study investigated the effect of equipment type on the effectiveness of manual ventilation and the ability to perform SLI using an intubated manikin model. This study was reviewed and approved by the Ethics Committee of the School of Medicine, University of Sao Paulo. Data were collected during the IV International Symposium on Neonatal Resuscitation of the Brazilian Society of Pediatrics held in São Paulo, Brazil.

From a total of 1,111 symposium participants, 114 individuals volunteered to participate in the study, of whom 54 were physicians trained as instructors in the Neonatal Resuscitation Program of the Brazilian Society of Pediatrics (instructor group), and 60 were physicians participating in the symposium who were not instructors (non-instructor group). Four individuals were excluded from the instructor and non-instructor groups (3 and 1 individuals, respectively) due to data acquisition failures, leaving 51 and 59 individuals, respectively, for analysis of respiratory mechanics. After respiratory mechanics data collection, 13 additional individuals were excluded from each group because they did not know how to perform the SLI maneuver. Thus, 38 individuals remained in the instructor group and 46 in the non-instructor group for the analysis of sustained inflation maneuvers with both equipment types.

All individuals included in the study were pediatricians who provided direct care to newborns in the delivery room. All participants were asked to ventilate the manikin and to perform the SLI maneuver without any prior explanation or demonstration of the correct method.

Using a standardized interview questionnaire, data were collected regarding the characteristics of the study participants, including age, gender, years since graduation, years since completion of residency, whether they were a Brazilian Society of Pediatrics Neonatal Resuscitation Program Instructor, years of experience as a program instructor, whether they held the title of Specialist in Neonatology, whether they worked in a neonatal intensive care unit (ICU), length of experience in neonatology, workplace, type of hospital in which they worked in the resuscitation room (public or private hospital), whether they worked in the resuscitation room of a university hospital, and the approximate number of newborns treated in the resuscitation room within the previous month.

### Manual ventilation

For the neonatal resuscitation scenario, a neonatal manikin (340 RN, Simulacare, São Paulo, SP, Brazil) was used, which is equivalent to a newborn weighing approximately 2,500 g. The manikin was intubated with a tracheal tube number 3.0 (Hi-Lo Oral/Tracheal Tube Cuffed, Intermediate, Murphy Eye, 4.3 mm OD, Covidien^™^, Mansfield, MA, USA), and the cuff was tested to confirm that the system had no air leaks.

The following scenario was described for each participant: "term newborn with an approximate birth weight of 2,500 g, asphyxiated, intubated and ready for manual ventilation. Ventilation should be performed for 3 min, and Vt should be evaluated based on the manikin's chest expansion." Following this explanation, each individual ventilated the manikin for two periods of 3 min each. During each period, the individual used either the T-piece (Babypuff^®^, Fanem LTDA, São Paulo, SP, Brazil) or the neonatal SIB with a 40-cmH2O pressure relief valve, with a capacity of 300 mL and no PEEP valve (Lifesaver^®^ Disposable Neonatal Manual Resuscitation Bag, Teleflex Medical, Research Triangle Park, NC, United States). The first equipment to be used by each individual was randomly determined, and the operator was blind to the recording of data related to respiratory mechanics. Data were recorded for a period of 3 min for each device. For ventilation using the T-piece, each individual chose the PIP and PEEP values according to their preference. A predetermined gas flow of 7 L/min was used. Respiratory rate (RR), Ti and expiratory times were also chosen by the participant. For ventilation using the SIB, RR and PIP values were also freely chosen by the operator.

### Sustained inflation maneuver

**Fig 1:**
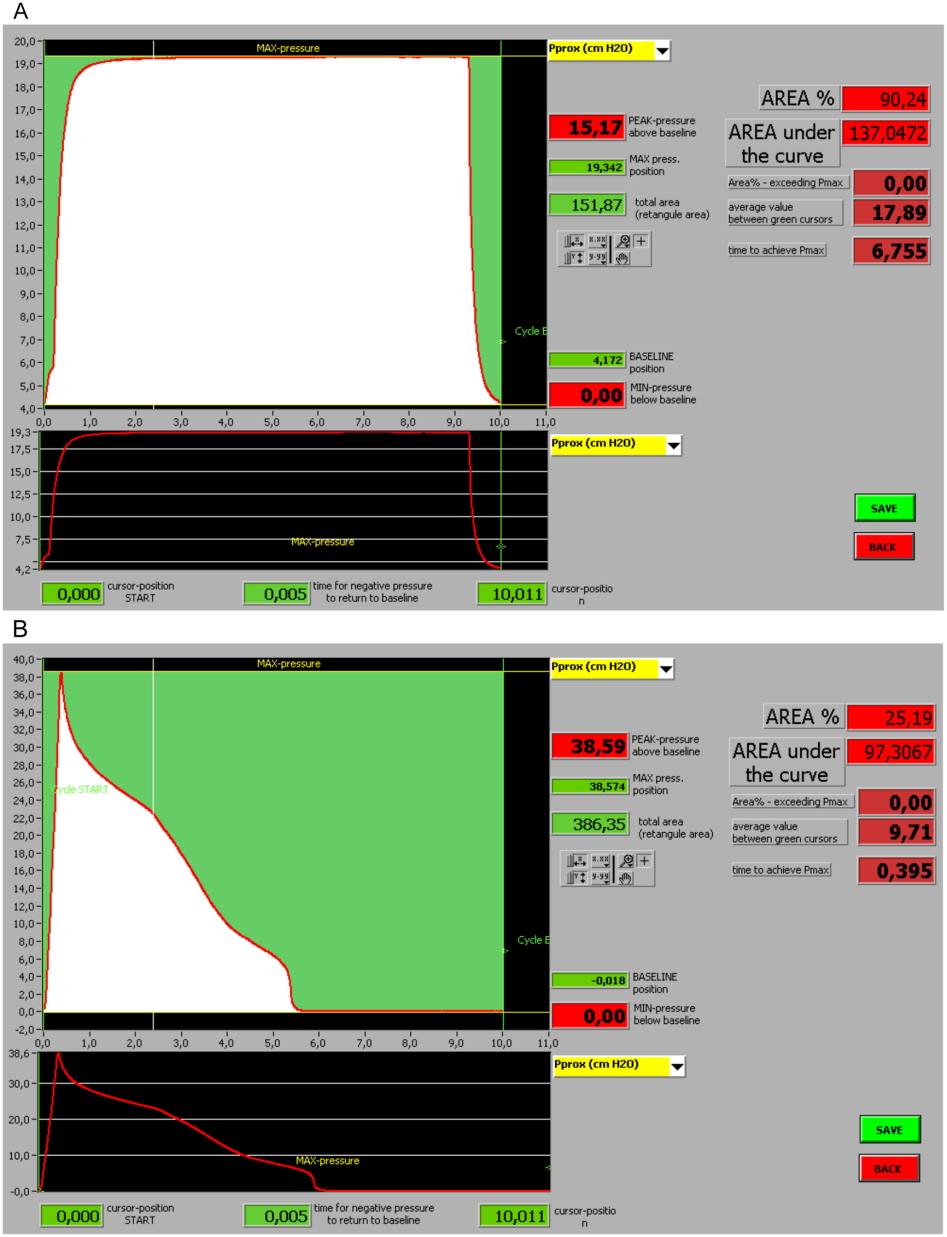
**(A)** Image showing the data acquisition screen of an SLI performed with a T-piece. **(B)** Image showing the data acquisition screen of an SLI performed with an SIB.

At the end of the ventilation period, each individual was asked to perform an SLI maneuver for a period of 10 seconds at a pressure of 20 cmH_2_O. To compare the effectiveness of the SLI maneuver, between both types of equipment and groups of individuals, software specifically developed for this purpose was used. This software allowed us to analyze the maximum pressure (P Max) obtained during the SLI maneuver; the area under the pressure-time curve (AUPTC) representing the capacity of each type of equipment to maintain SLI pressure during the 10-s period; the mean airway pressure (PMn) during the 10-s period; the time needed to obtain maximal inspiratory pressure (T Max); and the PEEP (Fig 1).

### Acquisition of respiratory mechanics data and analysis of results

During manual ventilation, the manikin was connected to a computerized data acquisition system. Ventilatory pressure was read using a pressure sensor (Validyne^®^, model DP45-24), and volume was calculated with a pneumotachograph (Hans Rudolph^®^ Inc., Kansas City, USA). Both of the above systems were calibrated and connected to data acquisition software (Lab VIEW^®^ 5.1, National Instruments, São Paulo, SP, Brazil) specifically developed for this purpose (RA Electro Systems LTDA, Campinas, Brazil). The ventilatory mechanics parameters PIP, PEEP, Vt, minute volume, RR, Ti and expiratory time were continuously assessed.

A direct comparison between the two types of equipment was performed for each respiratory mechanics and SLI parameter, followed by an analysis of equipment effectiveness, taking into account the education and training of the participants (instructor group or non-instructor group).

### Statistical analysis

Continuous data were compared using Student’s t test (comparison between types of equipment) and one-way analysis of variance (ANOVA) with the Student-Newman-Keuls post-hoc test (comparison between groups of individuals). The Mann-Whitney test or ANOVA based on ranks was used for nonparametric data. Categorical variables were analyzed using the chi-squared or Fisher's exact test, as appropriate. The statistical significance level was 0.05. The calculated sample to detect a difference in Vt of 1.0 mL between the groups was at least 25 subjects in each group, considering a standard deviation of 1.2 mL, a test power of 0.8 and a significance level of 0.05.

## Results

A comparison between the SIB and T-piece regarding the effectiveness of the pulmonary ventilation versus the SLI maneuver is shown in Table 1. The use of the T-piece resulted in lower Vt and PIP values, with increased PEEP and Ti. During the SLI maneuver, use of the T-piece resulted in a greater AUPTC, higher mean airway pressure during the 10-s period and longer time needed to reach a T Max of 20 cmH_2_O when compared with the SIB.

**Table 1:** Comparison between the SIB and T-piece regarding the effectiveness of pulmonary ventilation and SLI maneuver. Values are expressed as the median (interquartile range).

	T-piece	Self-inflating bag	P
**Manual ventilation**	(n = 110)	(n = 110)	
Vt (mL)	21.3 (9.1)	29.2 (14.0)	< 0.001
PIP (cmH_2_O)	19.9 (1.6)	21.1 (7.9)	< 0.001
PEEP (cmH_2_O)	5.1 (0.3)	0.0 (0.0–0.0)	< 0.001
Ti (seconds)	1.0 (0.9)	0.5 (0.2)	< 0.001
**Sustained inflation**	(n = 84)	(n = 84)	
P Max (cmH_2_O)	20.3 (0.5)	23.6 (10.6)	< 0.001
AUPTC (cmH_2_O/sec)	196.5 (10.9)	60.2 (68.8)	< 0.001
PMn (cmH_2_O)	19.7 (0.9)	6.4 (8.1)	< 0.001
T Max (seconds)	2.3 (0.9)	1.0 (0.8)	< 0.001

Vt, tidal volume; PIP, inspiratory pressure; PEEP, positive end-expiratory pressure; Ti, inspiratory time; P Max, maximum pressure obtained during the SLI maneuver; AUPTC, area under the pressure-time curve; PMn, mean airway pressure during 10 s; T Max, time to reach PIP.

Table 2 describes the characteristics of the professionals who participated in the study (S1 Data). There were differences between the two groups regarding years since graduation, years since completion of residency, and length of experience in neonatology.

**Table 2:** Characteristics of neonatal resuscitation professionals. Instructors are defined as individuals with specific training in neonatal resuscitation by the Brazilian Society of Pediatrics. Values are expressed as the mean ± standard deviation.

		Instructors	Non-Instructors	P
N		51	59	
Age (years)		46.8 ± 6.5	41.2 ± 10.6	< 0.01
Male gender n (%)		14 (27.5)	14 (23.7)	0.820
Time since graduation (years)		22.9 ± 6.9	16.5 ± 10.4	< 0.01
Time since completion of residency (years)		19.8 ± 7.3	12.4 ± 11.0	< 0.01
Experience in neonatology (years)		18.0 ± 7.2	11.8 ± 9.7	< 0.01
Title of specialist in neonatology		37 (72.5)	28 (47.5)	0.013
Works in NICU n (%)		48 (94.1)	53 (89.8)	0.639
	Public hospital n (%)	41 (80.4)	41 (69.5)	0.276
Workplace	Private hospital n (%)	30 (58.8)	35 (59.3)	0.888
	University hospital n (%)	21 (41.2)	35 (59.3)	0.088
Approximate number of newborns treated in previous month		30.6 ± 44.1	31.5 ± 29.5	0.403
Prior use of T-piece (%)		21 (41.2)	20 (33.9)	0.555

NICU: neonatal intensive care unit

### Respiratory mechanics

The Vt, Ti and PIP variables were used to evaluate the effectiveness of manual ventilation according to professional experience because these variables can have deleterious effects on lung development if improperly set (S2 Data).

**Fig 2:**
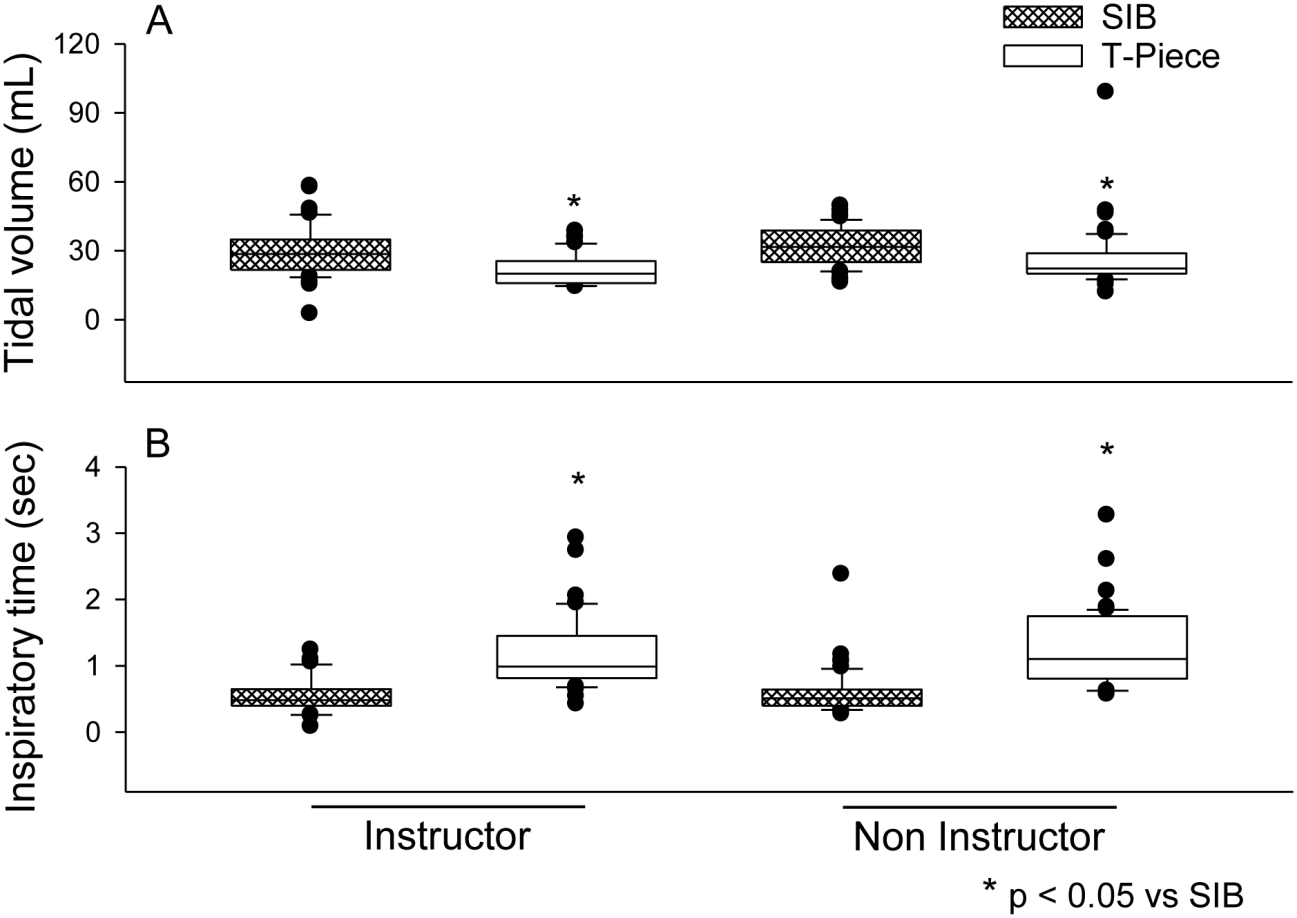
**(A)** Vt values according to the training of neonatal resuscitation professionals and type of equipment used for ventilation. **(B)** Ti values according to qualification and type of equipment used in neonatal ventilation.

A difference in Vt was found between the types of equipment, with medians (interquartile ranges) of 28.5 (12.6) mL and 20.1 (8.4) mL for the SIB and T-piece in the instructor group and 31.6 (14) mL and 22.3 (8.8) mL for the SIB and T-piece in the non-instructor group, respectively. There were no differences in Vt observed between groups (Fig 2A).

There was no difference in PIP between the groups. The median (interquartile range) for the SIB in the instructor group was 20.8 (7.6) cmH_2_O compared to 19.9 (1.2) cmH_2_O for the T-piece. The medians in the non-instructor group were 21.1 (8.2) cmH_2_O and 19.8 (1.7) cmH_2_O for the SIB and T-piece, respectively. There was no interaction between equipment type and level of education and individual training in relation to the generated PIP.

Similarly, the Ti differed between the two equipment types when analyzed in both groups of professionals. For the SIB and T-piece, the respective figures were 0.5 (0.2) vs. 1.0 (0.6) seconds for the instructor group and 0.5 (0.2) vs. 1.1 (0.9) seconds for non-instructor group, but no significant differences in this parameter were observed between the two groups of professionals. The interaction between the effect of equipment type and professional training on Ti was not significant (Fig 2B).

### Sustained inflation

**Fig 3:**
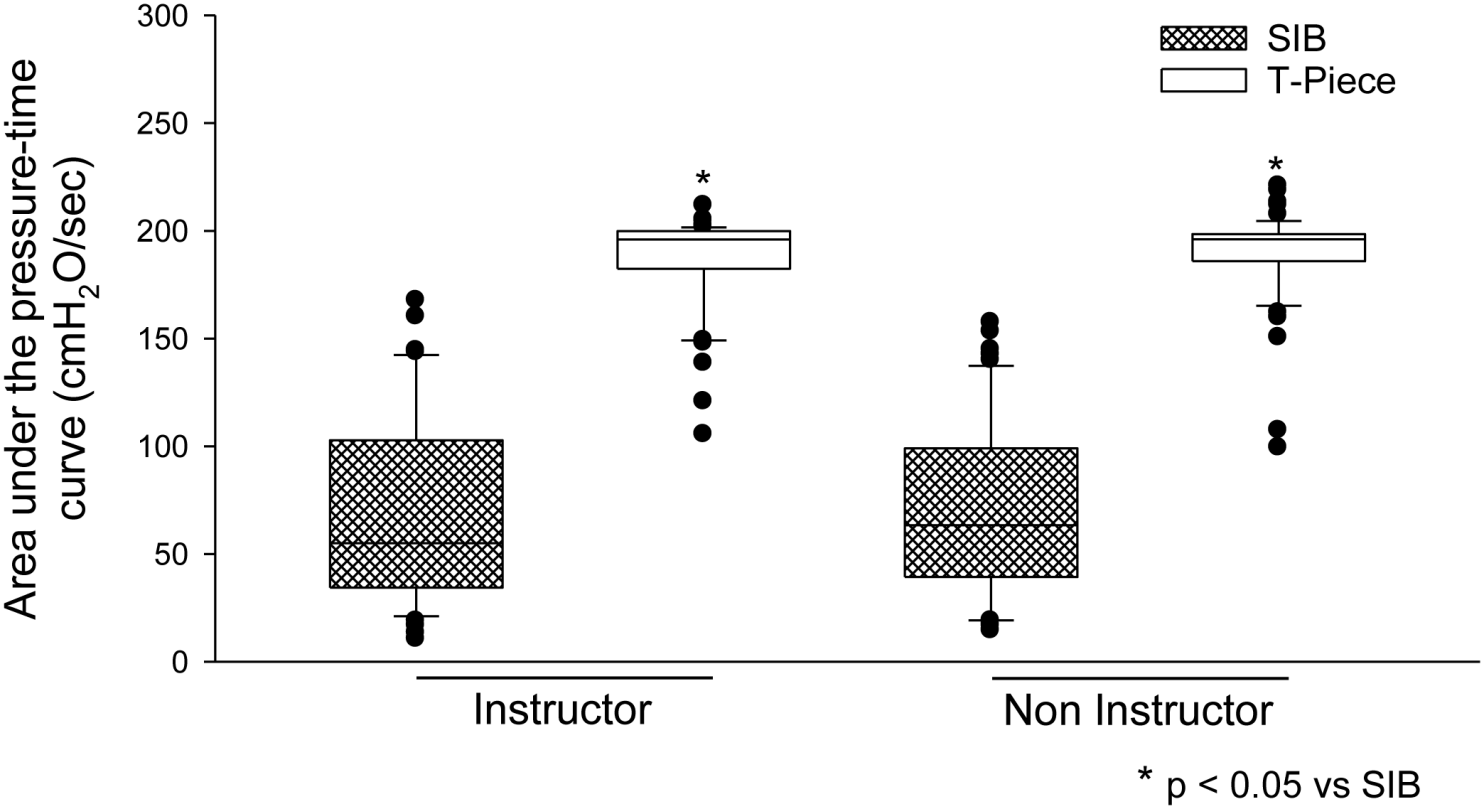
Ability to maintain sustained PIP during the 10-s procedure (measured by the area under the pressure-time curve).

**Fig 4:**
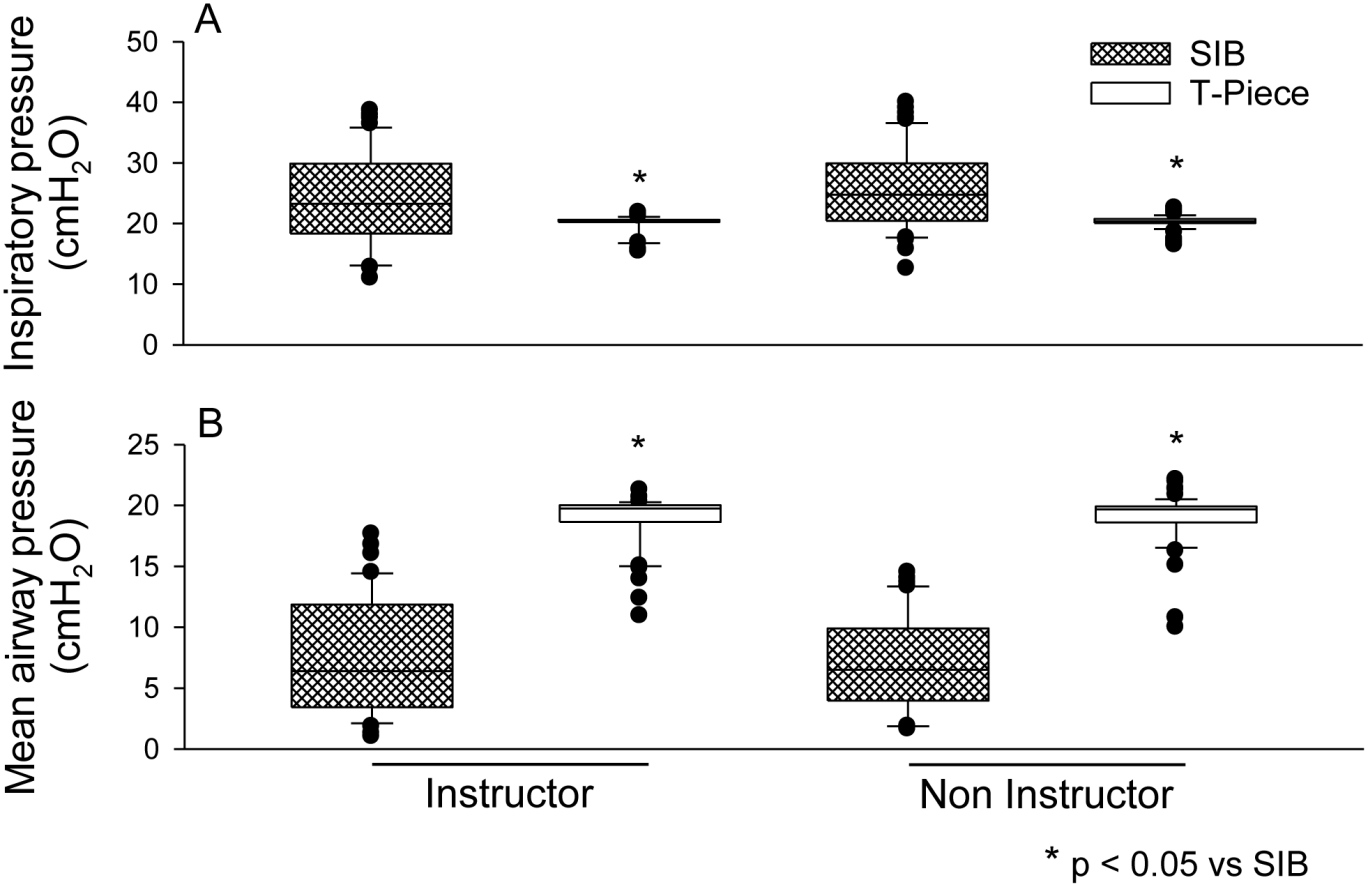
**(A)** Maximal inspiratory pressure values attained during SLI. **(B)** Mean pressure values between the beginning and end of the SLI procedure.

In the two groups of professionals, the operator's ability to maintain a target pressure for the 10-s SLI was improved with the use of the T-piece compared with the SIB (S3 Data). The median (interquartile range) of AUPTC for the SIB in the instructor group was 55.0 (66.7) cmH_2_O/sec compared to 195.9 (16.3) cmH_2_O/sec for the T-piece. The medians in the non-instructor group were 63.3 (56.3) cmH_2_O/sec and 196.0 (12.4) cmH_2_O/sec for the SIB and T-piece, respectively (Fig 3). Similarly, PIP applied with the SIB was greater than that applied with the T-piece. The median (interquartile range) for the SIB in the instructor group was 23.1 (11.4) cmH_2_O compared to 20.3 (0.4) cmH_2_O for the T-piece. The medians in the non-instructor group were 24.6 (9.2) cmH_2_O and 20.2 (0.7) cmH_2_O for the SIB and T-piece, respectively (Fig 4A). The PMn (measured between the beginning and the end of the 10-s procedure) was higher and more constant with the use of the T-piece in both groups. The median (interquartile range) for the SIB in the instructor group was 6.4 (8.3) cmH_2_O compared to 19.7 (1.3) cmH_2_O for the T-piece. The medians in the non-instructor group were 6.5 (5.5) cmH_2_O and 19.6 (1.3) cmH_2_O for the SIB and T-piece, respectively (Fig 4B).

## Discussion

In this study, we investigated the magnitude of the differences in respiratory mechanics variables observed between the SIB and T-piece during manual ventilation and SLI. We found significant differences between the equipment types in the provision of Vt and Ti during manual ventilation, with the T-piece providing more stable values. Similarly, Dawson et al. found PIP, PEEP and Vt to be more constant and reliable with the use of the T-piece [19].

Although the use of high Vt is harmful to the newborn’s lungs in manual ventilation, this parameter is not usually monitored during the neonatal resuscitation process [1]. Even at the start of ventilation using a Vt considered to be protective, there is early activation of the inflammatory response and expression of lung injury markers [20]. Indeed, we detected a significant clinical difference in Vt values between the two types of equipment, with a Vt value that was 7.6 mL greater when using the SIB. In view of the fact that a Vt in the 4 to 6 mL/kg range is recommended to minimize lung injury in newborns, the values measured suggest increased risk of lung injury when using the SIB.

We also detected differences in Vt between the two types of equipment, independent of education level and training in resuscitation. Roehr et al [16] reported lower Vt values in cases where SIB operators were more experienced, although Vt values were low when using the T-piece regardless of the experience level. However, we found higher Vt values compared to those of Roehr et al with both types of equipment, with the highest values obtained with the SIB. Thus, Vt should be monitored regardless of experience or equipment used. Furthermore, another study demonstrated a wide variation in Vt using the T-piece irrespective of the operator's experience level [21].

However, some authors have found no difference in PIP provided by experienced professionals between the compared equipment types [22]. We also found no differences in PIP magnitude between the equipment types and subject training levels. However, we detected greater variability in PIP when using the SIB. These findings are similar to those of other studies wherein the use of the SIB resulted in greater variability in PIP [15,16,22,23], even for more experienced operators. There was also greater variability in Vt, but less variability in PIP with the use of the T-piece [16]. Ti is controlled by the operator and is therefore subject to variation. In particular, a longer Ti is associated with a less experienced operator [21]. In another study conducted on a manikin, the Ti using the SIB was influenced by the operator's experience level but was within the limits of statistical significance. The same result was not observed for PIP, Vt or Ti when using the T-piece [15]. We found higher Ti values using the T-piece, with a magnitude of difference of 0.63 seconds. This difference is needed to obtain adequate alveolar pressure due to the increased time constant of the T-piece, determined by the expiratory valve resistance, which explains why ventilation with the T-piece using excessively short Ti can result in alveolar pressure that is lower than desired with reduced Vt.

We also evaluated the equipment’s capacity to maintain SLI during a 10-s period. To this end, we first measured the AUPTC over time for both devices. Next, we determined the area when the SIB was used relative to that observed with the use of the T piece. We found that the mean pressure between the beginning and the end of the 10-s procedure was 2.6 times higher for the T-piece, indicating a tendency toward greater effectiveness in lung aeration. Klingenberg et al [14] reported a higher mean pressure with the use of the T-piece and a lower mean pressure with use of the SIB. The SIB does not provide sufficient airflow to maintain effective SLI, and even with more experienced operators, any loss of air leads to a drop in PIP [24].

Furthermore, we found that in SLI, use of the T-piece resulted in a PIP within the target value and higher efficiency in maintaining this pressure throughout the proposed time period, as demonstrated by an AUPTC that was 1.7 times greater (169.4%) than that of the SIB. Similarly, Klingenberg et al [14] found that PIP was more consistent and stable over 10 seconds of SLI with the T-piece when compared to the flow-inflating bag, whereas SLI could not be maintained longer than 3 seconds when using the SIB, irrespective of the operator’s experience.

In testing different SIB models, other authors found that only one model achieved SLI levels similar to those provided by the T-piece [25]. Other SIB models did not support inflation of more than 15 seconds, which is well below the target value of 30 seconds [23]. This finding was also true of the SIB model chosen for our study, which did not reach the pressure target value during the 10-s period.

The decision to ventilate an intubated manikin instead of using face mask ventilation was based on the possibility of the occurrence of leaks around a face mask, which could result in misinterpretation of the data. It has been previously shown that even individuals with a high degree of training and experience in manual ventilation with a face mask have great difficulties in assessing the occurrence of leakage around the face mask without the aid of monitoring of respiratory mechanics [26], which could adversely affect the interpretation of the results.

This study developed a new and useful tool for evaluating the effectiveness of the SLI maneuver, based on the calculation of the AUPTC. This tool not only evaluates the PIP obtained but also correlates this pressure with the time over which it was maintained. This tool is useful for evaluating different equipment types used to perform SLI and allows a more detailed evaluation of the operators responsible for the maneuver. To our knowledge, this is the first time the use of this technology has made it possible to evaluate an SLI maneuver in such detail.

Nevertheless, our study had limitations. First, manual ventilation on a manikin cannot be compared to ventilation during actual resuscitation, particularly with regard to the stress associated with the resuscitation of an asphyxiated newborn and the changes to pulmonary mechanics that occur with time. However, for the circumstances described in this study, the observed differences are most likely comparable to those observed in a real situation. Second, participants were recruited from a national neonatal resuscitation conference; therefore, it is likely that the difference in experience between the individuals studied was lower than might be obtained in other settings because the non-instructor group consisted of individuals with greater interest in professionally updating their knowledge of neonatal resuscitation and most likely had greater overall knowledge of the subject.

## Conclusion

The authors conclude that manual ventilation using the T-piece results in lower Vt values and higher Ti values than those provided by the SIB, regardless of the operator’s level of training. Furthermore, the T-piece enables greater effectiveness in the SLI maneuver, represented by the maintenance of target pressure for the desired period and a higher mean airway pressure in comparison to that provided with the SIB. This improved performance is also independent of the equipment operator's training level. Thus, we conclude that our new analytic tool based on the AUPTC is useful because it allows measurable numerical differentiation between different equipment types and techniques when performing the SLI maneuver.

## Supporting Information

S1 Data Characteristics of the professionals who participated in the study. (PDF)

S2 Data Data used for the analysis of respiratory mechanics. (PDF)

S3 Data Data used for the analysis of sustained inflation maneuvers. (PDF)
