# Supplementary material for: Manual Ventilation and Sustained Lung Inflation in an Experimental Model: Influence of Equipment Type and Operator’s Training
Source: PLoS One. 2016 Feb 9;11(2):e0148475. doi: 10.1371/journal.pone.0148475 (PMC4747546; doi:10.1371/journal.pone.0148475)
Supplement: S1 Data — (PDF) [file pone.0148475.s001.pdf]

Order	Group	Age	Gender	Time since graduation (years)	Time since completion of residency (years)	Experience in neonatology (years)	Title of specialist in neonatology	Time as an instructor
1	I	48	M	25	22	22	S	13
2	I	48	F	24	22	20	S	11
3	I	45	M	22	20	20	N	4
5	I	44	F	20	16	15	S	14
7	I	34	M	9	6	6	S	1
8	I	47	F	23	20	20	S	15
9	I	46	F	21	18	18	N	1
10	I	49	M	23	20	20	N	3
12	I	42	F	17	14	13	S	10
13	I	47	F	24	21	21	S	14
14	I	51	F	28	24	20	S	15
15	I	59	M	36	34	34	S	16
16	I	48	M	25	23	23	S	10
17	I	52	M	28	25	25	S	15
18	I	46	F	23	20	20	S	15
19	I	56	F	32	28	28	S	15
20	I	59	F	35	32	31	S	16
21	I	57	M	34	32	23	S	15
22	I	47	M	24	20	20	S	17
24	N	24	F	1	0	0,5	N	0
25	N	46	F	22	18	19	S	0
26	N	45	M	20	9	10	N	0
28	N	31	F	5	1	1	S	0
29	N	40	F	16	13	13	S	0
30	N	34	M	10	7	10	S	0
31	N	31	F	8	4	4	S	0
32	N	32	F	6	0	3	S	0
33	N	66	F	38	36	38	N	0
34	N	37	F	13	10	3	N	0
35	N	34	F	9	6	9	S	0
36	N	33	F	8	6	5	N	0
37	N	53	F	29	26	0	N	0
38	N	36	M	9	5	6	S	0
39	I	40	F	16	13	15	S	7
40	N	29	F	5	3	3	S	0
41	N	29	F	5	0	2	N	0
42	N	34	F	5	1	3	N	0
43	N	34	F	10	6	6	N	0
44	N	48	M	23	21	21	N	0
45	N	33	M	8	1	3	N	0
46	N	53	M	29	27	20	S	0
47	I	44	F	20	15	17	S	13
48	N	38	F	14	10	10	S	0
50	I	52	F	27	25	25	S	6
52	I	45	F	19	15	15	S	5
53	I	52	F	29	27	18	S	8
54	I	42	F	19	17	10	S	10

58	N	38	F	11	7	7	S	0
59	N	41	F	18	10	10	N	0
60	N	47	F	23	19	19	S	0
61	N	28	F	4	1	1	N	0
62	I	33	F	8	3	5	S	5
63	N	29	F	5	1	3	N	0
65	I	43	M	20	17	17	S	0,5
66	I	46	M	22	19	18	S	2
68	N	43	F	18	0	8	N	0
69	I	45	F	22	18	18	S	15
70	N	31	F	7	5	5	S	0
71	I	52	M	27	25	15	N	2
72	I	38	F	12	8	8	N	1
73	N	56	F	31	27	27	N	0
74	I	53	F	31	26	26	N	2
75	N	30	M	4	0,8	0,8	N	0
76	N	33	F	5	0,8	0,8	N	0
77	I	44	F	20	17	17	S	13
78	I	56	F	32	30	25	N	1
79	N	32	F	6	1,8	3	N	0
80	N	30	F	6	0	1	N	0
81	N	29	F	4	0	1,5	N	0
82	I	56	F	33	30	15	S	1
83	N	44	F	19	16	16	S	0
84	N	62	M	33	0	33	N	0
85	I	38	F	12	8	8	S	5
86	I	31	F	9	6	6	S	1
87	I	52	F	29	27	26	S	15
88	N	49	M	25	22	22	N	0
89	N	35	M	10	7	8	S	0
90	N	48	M	23	21	15	S	0
91	N	64	M	37	35	20	S	0
92	N	40	F	14	10	10	N	0
93	N	50	F	27	24	24	S	0
94	N	31	F	3	1	1	N	0
95	I	48	F	26	22	22	N	0,5
96	N	54	M	29	26	23	S	0
97	N	54	F	30	28	24	N	0
98	I	44	F	20	17	17	S	15
99	N	38	F	16	12	12	S	0
100	I	35	F	12	6	10	S	2
101	I	41	F	16	13	14	S	10
102	N	52	F	20	27	24	S	0
103	N	52	F	29	26	25	S	0
104	N	64	F	37	35	10	N	0
105	I	49	F	26	23	23	S	15
106	N	41	F	17	14	14	S	0
107	I	44	F	20	17	12	N	12
108	N	42	F	23	19	13	N	0
109	N	37	F	13	9	9	S	0
110	I	56	F	31	28	30	N	2
111	N	55	F	34	31	31	S	0

112	N	56	M	168	32	30	N	0
114	I	51	M	24	22	22	N	15
115	I	45	M	19	16	8	N	2
116	N	48	F	23	19	18	N	0
117	N	34	F	10	7	7	S	0
118	I	47	F	23	21	21	S	16
119	N	40	F	22	18	18	S	0
120	I	40	F	14	11	0	N	4
121	N	38	F	15	10	10	N	0
122	I	48	F	24	21	10	N	11
123	I	53	F	32	28	28	S	15

Order	ICU	Public hospital	Private hospital	University hospital	Number of newborns treated in previous month	Use of T-piece
1	S	S	S	N	6	S
2	S	S	S	S	30	N
3	S	S	S	S	50	S
5	S	N	S	N	30	S
7	S	S	S	S	100	N
8	S	S	S	S	20	N
9	S	S	S	S	15	N
10	S	S	S	S	40	N
12	S	N	S	N	50	S
13	S	S	S	S	20	S
14	S	S	S	N	35	N
15	S	S	N	S	0	N
16	S	S	S	N	20	S
17	S	S	N	S	10	S
18	S	S	N	S	5	N
19	S	S	N	S	2	S
20	S	N	N	S	5	S
21	S	N	S	S	2	S
22	S	S	S	S	60	S
24	S	N	S	S	20	N
25	S	N	S	N	10	N
26	S	S	S	N	50	N
28	S	S	S	N	50	S
29	S	S	S	S	15	N
30	S	N	N	N	100	N
31	S	S	S	S	20	S
32	S	S	S	S	80	S
33	S	N	N	N	40	N
34	S	N	S	N	5	N
35	S	N	S	N	10	S
36	N	S	N	N	12	N
37	N	N	N	N	0	N
38	S	S	S	S	15	S
39	S	S	S	S	20	S
40	S	S	S	S	15	S
41	S	S	S	S	125	S

42	S	S	S	S	40	S
43	S	N	S	N	40	N
44	S	S	N	N	60	N
45	S	S	S	S	20	N
46	S	S	S	N	30	N
47	S	S	N	S	0	N
48	S	S	N	S	32	N
50	S	N	S	N	12	S
52	S	S	N	N	10	S
53	S	S	N	S	10	N
54	S	S	S	S	30	S
58	S	S	S	N	40	N
59	S	N	N	N	15	N
60	S	S	S	S	50	S
61	S	S	S	N	50	S
62	S	S	N	N	16	S
63	S	S	S	N	100	S
65	S	S	N	S	4	S
66	S	S	N	S	10	S
68	S	S	N	N	8	S
69	S	S	S	S	20	S
70	S	N	S	N	15	S
71	S	N	S	S	20	N
72	S	S	S	S	15	N
73	S	S	N	N	32	N
74	S	S	S	N	30	N
75	S	S	N	N	30	N
76	S	S	S	N	50	N
77	S	S	S	S	35	S
78	S	S	S	N	60	N
79	S	S	N	S	30	N
80	S	N	N	N	20	N
81	N	S	N	N	10	N
82	S	S	S	N	64	N
83	S	N	S	S	12	N
84	S	S	N	N	10	N
85	S	S	N	N	40	N
86	S	S	N	S	20	N
87	S	S	N	S	50	N
88	S	S	S	N	5	N
89	S	N	N	N	1	N
90	S	S	N	N	30	S
91	S	N	S	N	0	N
92	S	S	N	N	24	N
93	S	S	S	S	12	S
94	S	S	N	S	20	S
95	S	S	N	N	10	N
96	S	S	S	S	150	N
97	N	S	S	N	56	N
98	S	S	N	N	30	N
99	S	S	S	S	15	N
100	S	N	S	S	10	N

101	S	S	N	S	20	N
102	S	N	S	N	30	N
103	S	N	S	N	4	N
104	S	S	N	N	40	N
105	S	S	S	N	40	N
106	S	S	S	S	30	S
107	S	S	N	N	300	N
108	S	S	S	S	40	S
109	S	S	N	S	30	N
110	N	N	N	N	30	N
111	S	N	N	S	25	S
112	N	N	N	N	8	N
114	S	S	S	N	30	N
115	S	S	S	N	40	N
116	N	S	S	N	10	N
117	S	S	N	S	10	N
118	S	S	S	S	80	N
119	S	S	S	S	28	S
120	N	S	N	S	1	N
121	S	S	N	S	30	N
122	N	N	N	N	0	N
123	S	N	S	N	3	S
