# Supplementary material for: Manual Ventilation and Sustained Lung Inflation in an Experimental Model: Influence of Equipment Type and Operator’s Training
Source: PLoS One. 2016 Feb 9;11(2):e0148475. doi: 10.1371/journal.pone.0148475 (PMC4747546; doi:10.1371/journal.pone.0148475)
Supplement: S2 Data — (PDF) [file pone.0148475.s002.pdf]

Order	PIP-A x	PIP-A sd	Vt-A x	Vt-A sd	PIP-T x	PIP-T sd	PEEP-T x	PEEP-T sd
1	21,43	3,86	30,9	6,22	20,55	0,28	7	0,08
2	24,18	2,26	37,33	3,58	20,71	0,17	7,08	0,04
3	37,78	1,49	56,4	0,7	20,25	0,08	5,7	0,1
5	22,43	5,03	41,35	6,43	19,96	0,2	4,93	0,05
7	16,86	2,33	25,2	3,33	19,61	0,31	5,63	0,12
8	14,76	2,75	22,46	1,07	20,61	0,11	5,3	0,06
9	24,1	2,58	28,15	3,48	19,76	0,23	5,08	0,02
10	30,56	3,75	50,45	2,55	19,63	0,1	5,48	0,11
12	24	1,78	27,58	1,13	20,05	0,08	5	0
13	14,88	2,84	19,46	2,39	19,9	0,1	5,23	0,25
14	10,21	4,69	15,08	1,44	16,1	0,2	4,9	0
15	25,18	4,58	39	6,33	19,5	0,34	5,3	0
16	15,43	1,57	21,23	5,63	20,08	0,22	5,11	0,04
17	23,65	1,73	42,05	5,42	19,8	0,45	3,4	0
18	10,9	6,24	15,41	9	19,31	1,58	5,1	0
19	19,91	3,74	32,76	6,49	19,78	0,04	4,81	0,07
20	24,58	1,56	37,31	3,61	19,98	0,31	3,95	0,1
21	22,8	1,86	41,13	4,62	20,36	0,19	5,15	0,08
22	14,18	1,09	22,71	1,81	19,31	0,38	4,9	0
24	17,3	1,8	20,1	5,14	16,63	0,08	5,2	0
25	16,71	1,24	26,58	3,77	16,16	0,1	4,98	0,04
26	29,6	2,39	41,95	4,01	18,21	0,16	5,1	0,06
28	27,66	1,36	39,31	1,2	19,91	0,11	5,16	0,05
29	27,08	3,04	35,68	2,54	19,83	0,08	5,1	0
30	19,18	2,04	29,35	1,46	27,55	0,77	5,25	0,15
31	22,53	2,09	31,66	2,38	18,65	1,83	5,78	0,73
32	29,96	4,42	48,53	6,88	21,38	0,21	5,18	0,11
33	15,18	1,91	17,45	2,46	20,83	0,22	6,11	0,11
34	18,66	3,37	22,96	5,65	15,23	0,1	5,15	0,05
35	27,18	3,36	43,89	4,91	14,88	0,04	4,88	0,07
36	25,28	3,59	47,2	9,1	30,23	0,26	5,3	0
37	26,98	3,16	51,51	6,19	20,81	0,27	5,1	0,1
38	25,31	1,51	36,94	4,75	24,86	0,05	5,4	0
39	17,66	0,38	30,35	2,5	18,9	0,1	5,4	0,16
40	17,61	2,29	26,66	1,97	19,75	0,05	5,11	0,04
41	16,01	0,98	23,99	3,21	20	0,1	5,3	0
42	23,5	2,25	38,67	4,9	19,43	0,2	6,1	0
43	19,08	2,32	32,63	4,24	18,78	0,04	6,23	0,16
44	34,25	1,17	44,96	0,41	21,95	0,23	5,06	0,05
45	24,33	1,26	34,65	1,32	18,35	0,08	5,2	0,06
46	17,21	1,06	27,37	1,88	16,8	0,08	5,21	0,04
47	25,65	2,81	42,45	5,73	16,8	0	5,1	0
48	19,1	4,03	28,43	7,15	19,56	0,41	5	0
50	24,61	2,06	37,45	4,63	20	0	5,2	0
52	19	2,44	31,49	2,84	20,28	0,04	5	0
53	17,5	4,04	19,78	7,67	21,26	0,47	4,85	1,16
54	26,73	3,21	47,9	3,72	20,46	0,15	5,21	0,04
58	24,66	2,16	20,79	1,03	19,88	0,11	4,9	0,06
59	25,03	1,69	21,77	3,86	20,36	0,08	5	0
60	31,15	2,5	32,86	2,17	19,98	0,04	4,98	0,04
61	24,83	1,16	35,53	2,71	19,33	0,12	4,91	0,04

62	27	2,89	26,98	2,8	16,15	0,08	5	0
63	23,66	4,67	22,09	5,68	20,31	0,09	4,71	0,04
65	17,83	2,22	14,36	1,97	19,83	0,4	4,9	0
66	14,66	1,59	21,95	2,74	16,26	0,42	3	0
68	29,58	4,48	44,23	1,63	20,76	0,1	5,16	0,08
69	20,36	2,73	29,22	2,32	20,65	0,05	5,88	0,04
70	20,46	1,78	25,5	1,58	20,11	0,22	5,15	0,05
71	23,95	2,52	29,92	1,13	16,38	0,07	5	0
72	21,1	4,17	31,72	3,25	20,06	0,12	5,18	0,04
73	11,46	3,46	17,3	4,09	20,1	0	5	0,06
74	13	2,44	14,24	1,1	20,21	0,09	5,55	0,28
75	14,16	2,63	23	3,89	20,1	0,14	5,2	0
76	15	4,47	25,38	4,57	20,1	0,24	5,2	0
77	19,61	1,49	27,24	2,1	19,9	0	5,2	0
78	21,91	2,41	28,84	2,37	20,2	0,24	5,31	0,04
79	25	2,28	34,6	4,7	20,4	0	5,48	0,2
80	16,2	2	24,98	4,48	19,41	0,04	5	0
81	26,08	1,85	39,48	2,76	16,61	0,04	4,95	0,05
82	15,05	1,9	22,53	2,15	15,5	0	5	0
83	25	4,85	37,43	4,33	12,86	0,08	2,2	0
84	20,66	4,22	33,06	7,95	16,3	0	5	0
85	19	3,09	24,91	3,65	19,5	0	4,15	0,05
86	21,73	2,25	27,34	1,67	15,21	0,04	4,33	0,05
87	16,26	1,42	23,51	0,68	19,9	0,06	5,1	0
88	21,13	1,5	29,39	2,26	19,26	0,38	5,1	0
89	19,96	1,67	26,11	3,22	19,9	0	5,1	0
90	14,53	1,57	21,27	2,22	19,66	0,18	5	0
91	21	2,52	29,27	4,84	20,58	0,07	5,46	0,21
92	16,28	0,04	4,66	0,04	18,1	0,59	4,66	0,04
93	15,15	2,01	22,5	3,13	17,96	0,27	5,11	0,09
94	20,46	1,42	33,85	3,09	20,3	0,15	5,16	0,05
95	19,41	1,68	30,18	5,88	20,55	0,08	5,06	0,05
96	20	2,22	33,4	2,49	20,66	0,05	5,1	0
97	27,9	2,39	42,01	3,23	20,4	0,06	5,25	0,12
98	26,7	2,36	35	2,9	18,83	0,08	4,21	0,04
99	18,85	1,24	27,8	3,16	17,1	0,12	5,25	1,19
100	12,96	2,22	18,93	1,87	16,3	0,18	4,75	0,36
101	18,56	2,81	24,75	4,14	14,73	0,56	4,8	0,1
102	30,5	3,01	41,45	2,29	19,68	0,09	5	0
103	20,3	1,95	33,35	0,9	19,95	0,18	5,03	0,05
104	18,85	3,99	24,15	5,22	19,01	0,04	5,01	0,04
105	26,33	7,22	39,47	3,12	21,95	0,12	4,43	0,27
106	27,33	5	37,5	5,14	20,33	0,08	5,2	0
107	20,81	2,63	30,36	2,46	19,88	0,58	8,05	0,51
108	23,66	1,36	32,66	1,27	19,88	0,04	5,1	0
109	22,33	4,5	31,39	2,89	20,06	0,08	5,08	0,04
110	20,16	4,11	30,46	5,04	20,21	0,09	3,03	0,05
111	27,56	4,42	34,87	3,47	20,15	0,05	5,18	0,04
112	26	5,86	39,29	8,29	20,41	0,09	5,43	0,05
114	31,33	2,8	53,94	6,39	16,4	0	4,96	0,08
115	28,33	5,6	46,09	5,63	20,26	0,22	6,3	0
116	19,81	3,41	33,49	5,14	20,5	0	5,11	0,04

117	15,66	3,07	23,72	4,65	18,65	0,37	5,1	0
118	29,16	3,43	41,61	4,28	20	0	5,1	0
119	27,5	4,96	39,63	5,05	18,46	0,05	6,26	0,05
120	20,83	3,65	28,89	8,46	16,18	0,09	5,3	0
121	14,7	0,55	22,42	3,09	19,51	0,26	5,2	0
122	37	0,89	57,72	0,92	20,35	0,05	5,3	0
123	15,58	3,63	21,54	4,64	19,95	0,29	5,1	0

Order	Vt-T x	Vt-T sd	PEEP	PIP
1	22,7	1,75	5	20
2	21,36	1,14	5	20
3	38,21	2,94	5	20
5	28,95	1,69	5	20
7	25,72	0,38	5	20
8	35,16	1,04	5	20
9	34,6	1,63	5	20
10	23,15	1,05	5	20
12	27,4	2,12	5	20
13	29,01	1,3	5	20
14	10,01	0,59	5	16
15	23,6	1,25	5	20
16	31,13	1,46	5	20
17	33,82	1,48	3	20
18	18,97	5,56	5	20
19	25,98	1,72	5	20
20	31,71	3,14	4	20
21	31	1,44	5	20
22	28,58	2,04	5	20
24	25,2	2,35	5	15
25	24,43	0,58	5	18
26	23,86	0,4	5	18
28	20,3	0,52	5	20
29	19,51	0,55	5	18
30	32,96	1,96	5	30
31	17,75	2,18	5	15
32	27,85	0,6	5	20
33	35,03	1,01	5	20
34	23,31	0,77	5	15
35	22,93	2,76	5	15
36	46,26	4,51	5	30
37	32,5	10,55	5	20
38	37,45	1,19	5	25
39	20,03	1,05	5	20
40	31,83	0,83	5	20
41	36,7	2,38	5	20
42	25,91	0,45	6	20
43	19,07	0,33	6	18
44	32,78	1,86	5	20
45	29,53	1,79	5	15
46	25,29	1,57	5	15
47	20,94	3,99	5	17
48	30,38	0,85	5	19
50	27,38	0,53	5	20

52	37,49	2,83	5	20
53	19,68	0,26	5	20
54	33,26	1,27	5	20
58	25,64	4,13	5	18
59	29,63	1,76	5	20
60	29,85	2,92	5	20
61	38,65	4,22	5	20
62	24,52	1,33	5	15
63	47,31	2,99	5	20
65	14,36	1,58	5	20
66	19,91	0,85	3	18
68	21,93	0,61	5	20
69	18,84	0,33	5	20
70	18,54	0,35	5	20
71	14,7	0,41	5	15
72	20,82	0,35	5	20
73	22,23	0,35	5	20
74	20,18	0,46	5	20
75	19,99	0,37	5	20
76	19,41	1,03	5	20
77	17,73	0,54	5	20
78	21,48	0,41	5	20
79	22,29	0,19	5	20
80	20,94	0,16	5	18
81	18,1	0,48	5	15
82	15,25	0,25	5	15
83	15,38	0,52	2	12
84	15,86	0,17	5	15
85	21,11	0,47	5	20
86	14,61	0,94	5	15
87	20,06	0,24	5	20
88	18,17	1,04	5	20
89	20,29	0,37	5	20
90	19,79	0,26	5	20
91	23,28	1,65	5	20
92	18,27	0,63	5	15
93	18,86	0,75	5	18
94	21,86	0,34	5	20
95	23,21	0,53	5	20
96	23	0,57	5	20
97	21,01	0,67	5	20
98	21,11	0,24	4	17
99	17,82	0,53	5	16
100	15,35	0,74	5	20
101	13,73	0,74	5	15
102	19,91	0,42	5	20
103	21,13	0,39	5	20
104	20,23	0,18	5	18
105	26,18	0,42	4	22
106	20,54	0,68	5	20
107	15,53	0,74	6	18
108	20,23	0,54	5	20

109	20,69	0,69	5	20
110	25,94	0,4	3	20
111	22,4	0,5	5	20
112	22	0,42	5	20
114	17,03	0,63	5	15
115	19,69	0,45	6	20
116	22,6	0,07	5	20
117	18,14	0,28	5	18
118	22,28	0,6	5	20
119	17,66	0,37	5	15
120	15,74	0,31	5	15
121	20,2	0,45	5	20
122	22,98	0,28	4	20
123	21,9	1,14	5	20
