# Supplementary material for: Manual Ventilation and Sustained Lung Inflation in an Experimental Model: Influence of Equipment Type and Operator’s Training
Source: PLoS One. 2016 Feb 9;11(2):e0148475. doi: 10.1371/journal.pone.0148475 (PMC4747546; doi:10.1371/journal.pone.0148475)
Supplement: S3 Data — (PDF) [file pone.0148475.s003.pdf]

Order	A PIP B	B PIP B	A PIP Max	B PIP Max	A AUC	B AUC	A mean	B mean
1	35,63	16,59	35,58	21,19	55,58	151,83	17,62	19,8
2	37,33	14,6	37,22	21,63	46,83	135,62	11,82	20,5
5	25,42	15,94	25,37	20,55	48,36	151,03	11,83	19,64
8	12,62	15,66	12,56	20,77	10,38	134,01	1,01	20,13
9	26,02	15,39	25,94	20,3	31,42	141,86	3,46	19,64
12	18,25	15,24	18,19	20,27	16,7	133,31	1,81	17,89
13	13,24	15,87	13,17	20,73	22,04	144,61	2,24	20
15	27,25	15,41	27,18	20,34	64,75	147,9	6,47	19,44
16	26,97	15,8	26,91	20,76	46,83	152,19	4,94	20,18
17	23,47	17,28	23,4	20,53	53,95	162,39	5,46	20,11
18	18,36	15,51	18,33	20,44	41,94	158,28	4,27	19,94
19		16,03		20,57		174,54		19,8
20	25,75	17,26	25,68	20,94	49,31	163,66	5,39	20,07
21	21,02	15,68	20,94	20,66	29,1	179,96	2,78	20,26
22	14,7	15,59	14,64	20,28	65,97	144,74	8,37	19,68
23		10,54		15,35		91,25		14,51
24		15,02		20,06		131,09		19,51
25	26,65	15,18	26,53	20,01	55,58	150,96	5,2	19,47
26								
28								
29	23,24	15,12	23,14	20,14	153,04	170,47	13,35	19,62
30								
31	32,15	15,29	32,14	20,45	157,31	140,61	13,66	18,89
32	24,29	17,16	24,18	22	47,07	164,98	6,31	21,36
33								
34	38,12	15,14	38,02	20,12	19,45	148,66	1,84	19,55
35	22,32	2,99	22,21	20,12	27,29	31,87	2,6	20,02
36								
37	23,15	17,61	23,06	22,51	53,94	166,72	5,64	21,73
38	28,95	17,5	28,86	22,5	38,65	185,03	3,92	22,08
39	17,6	15,36	17,53	20,27	28,75	146,33	3,12	19,5
40								
41	19,27	15,23	19,19	20,19	14,39	152,9	1,64	19,81
42	20,52	14,54	20,45	20,35	16,61	143,88	1,62	19,93
43	12,52	12,52	12,45	12,45	23,94	23,94	2,34	2,38
44	26,17	16,85	26,11	21,72	61,5	161,6	7,5	21,24
45	26,57	13,6	26,5	18,56	53,43	124,95	5,31	18,03
46								
47	32,14	8,74	32,07	17,18	61,97	87,36	6,19	17,14
48	25,61	15,43	25,55	20,35	40,59	138,02	4,08	19,82
50		15,57		20,47		154,35		19,89
52	25,8	15,65	25,74	20,47	35,32	149,23	3,3	20,08
53	21,75	16,83	21,63	21,78	64,65	160,26	6,5	21,12
54	20,62	15,36	20,55	20,28	13,16	146,08	1,25	19,53
58	24,75	15,63	24,67	20,62	25,56	136	2,51	18,54
59								
60	37,1	14,75	37,12	19,6	18,89	124,48	1,94	17,3
61								
63	30,67	15,71	30,61	20,61	16,9	141,16	1,65	19,04
65		15,46		20,32		146,86		18,58
68	39	16,05	38,83	21,06	73,44	149,48	8,05	20,72

69	29,86	16,21	29,75	21,2	120,4	156,4	12	20,7
70	18,43	15,64	18,32	20,91	58,86	151,4	5,81	20,48
71		12,04		16,94		116,61		16,61
72	29,81	15,32	29,73	20,28	126,01	145,52	12,57	19,58
73	17,45	15,48	17,36	20,23	46,69	149	4,6	19,75
74	18,78	15,65	18,69	20,64	45,71	150,08	4,51	20,06
75								
76	18,04	15,78	17,97	20,79	41,63	148,56	4,12	20,4
77	20,99	15,75	20,91	20,62	54,4	152,32	5,39	20,16
78		15,59		20,59		150,67		20,12
79								
80	19,86	15,58	19,8	20,39	76,93	151,71	7,77	20,03
81								
82	18,5	10,85	18,45	15,67	62,19	103,57	6,22	15,22
83	15,76	18,15	15,71	20,32	36,3	180,61	3,58	19,89
84								
85	20,56	16,36	20,53	20,21	143,44	160,02	14,46	19,87
86	29,7	14,8	29,69	20,41	144,33	143,82	14,42	20
88	25,64	15,14	25,61	20,15	105,13	133,77	10,53	19.68
89	17,57	15,37	17,54	20,24	65,22	136,49	7,12	19,85
90	23,25	15,19	23,32	20,04	117,73	141,3	11,94	19,37
91		15,66		20,77		145,6		19,87
92	28,96	11,87	28,94	16,38	92,93	107,67	9,34	15,63
93	27,07	16,33	27,05	21,31	134,21	159,112	13,1	20,9
94	17,87	15,86	17,84	20,84	18,58	148,79	1,85	20,05
95	38,59	15,86	38,57	20,8	97,3	154,59	9,71	20,38
96	19,11	16,09	19,11	20,98	140,53	144,98	14,56	20,51
97	32,01	15,77	32	20,6	72,35	144,89	7,59	19,98
98	30,93	15,17	30,91	19,34	87,96	137,04	8,87	17,89
99	21,64	12,86	21,64	17,37	120,99	123,8	12,07	16,95
100	16,76	12,42	17,05	16,82	118,42	120,9	12,12	16,48
101	15,45	10,59	15,44	15,48	102,03	99,12	11,09	15,22
102	25,59	15,37	25,59	20,13	112,49	149,11	11,27	19,72
103	19,95	15,41	19,94	20,22	69,14	148,22	6,95	19,73
104		17,45		21,36		165,49		20,64
106	26,65	15,9	26,58	20,86	92,48	155,84	9,19	20,56
107	37,87	12,82	37,8	20,36	120,76	123,71	12,12	20
108	25,97	15,74	25,9	20,52	44,19	152,31	4,4	20,1
109	32,6	15,64	32,5	20,52	117,51	151,32	11,69	20,06
110	10,94	17,61	10,88	20,48	28,84	147,8	2,84	17,67
111	20,43	15,36	20,36	20,32	70,68	148,22	7,01	19,83
112	23,63	15,39	23,67	20,6	40	147,53	4,05	20,03
113								
114	33,93	11,93	33,94	16,67	167,93	115,75	16,82	16,33
115	27,66	14,46	27,62	20,37	125,82	141,17	12,56	20,04
116	29,19	15,9	29,16	20,79	76,26	154,96	7,27	20,4
117		15,43		20,33		149,78		19,93
118	23,5	15,36	23,56	20,45	159,61	143,46	16,09	19,49
119	31,91	14,44	31,88	20,51	63,35	55,5	6,32	11,65
120	12,66	15,11	12,63	20,3	27,11	146,13	2,71	19,81
121	17,22	14,88	17,2	19,95	78,25	138,97	7,81	19,02
122	30,03	15,69	30,02	20,64	141,1	152,44	14,12	20,2

123	12,75	15,67	12,72	20,63	23,51	150,62	2,33	20,05
Order	A Tmax	B Tmax	A basel	B basel				
1	1,06	10,03	0	4,58				
2	0,6	1,2	0	7,03				
5	0,84	8,58	0	4,61				
8	0,52	9	0	5,12				
9	0,95	5,71	0	4,91				
12	1,65	0,96	0	5,03				
13	1,67	9,58	0	4,86				
15	1,1	5,26	0	4,93				
16	0,95	8,3	0	4,95				
17	0,55	8,29	0	3,46				
18	1,04	8,45	0	4,93				
19		11,19		4,54				
20	1,76	10,04	0	3,68				
21	0,96	11,98	0	4,97				
22	2,74	6,66	0	4,69				
23		9,76		4,81				
24		9,77		5,03				
25	0,64	10,28	0	4,83				
26								
28								
29	1,14	10,87	0	5,02				
30								
31	0,5	9,18	0	5,16				
32	0,88	8,8	0	4,84				
33								
34	0,37	10,3	0	4,96				
35	1,1	9,33	0	17,136				
36								
37	1,68	3,54	0	4,9				
38	1	2,83	0	5				
39	1,45	6,53	0	4,91				
40								
41	2,04	10,34	0	4,95				
42	0,66	6,83	0	5,81				
43	0,83	0,97	0	0				
44	2,4	1,66	0	4,87				
45	0,59	4,82	0	4,97				
46								
47	0,58	8	0	8,44				
48	1,08	9,72	0	4,92				
50		9,5		4,91				
52	0,72	4,45	0	4,83				
53	1,2	9,42	0	4,95				
54	0,91	2,3	0	4,92				
58	0,53	0,4	0	5				
59								
60	0,47	4,8	0,02	4,83				
61								
63	0,48	0,37	0	4,9				
65		0,29		4,85				

68	0,52	6,52	0	5,01
69	0,92	8,54	0	5
70	0,73	9,9	0	5,27
71		9,33		4,9
72	0,7	9,96	0	4,96
73	0,4	10,05	0	4,75
74	0,7	8,29	0	5
75				
76	0,86	9,3	0	5,01
77	0,52	7,9	0	4,87
78		9,38		5
79				
80	0,88	9,71	0	4,81
81				
82	0,44	6,72	0	4,82
83	0,51	5,83	0	2,17
84				
85	0,5	7,43	0	3,85
86	0,66	3,52	0	5,61
88	0,97	6,05	0	5
89	0,39	8,3	0	4,88
90	0,78	6,66	0	4,86
91		6,77		5,12
92	0,72	7,29	0	4,51
93	0,53	9,71	0	5
94	0,75	9,19		5
95	0,39	7,35	0	4,94
96	2,91	8,18	0	4,89
97	0,72	6,38	0	4,81
98	0,63	6,75	0	4,17
99	1,01	8,17	0	4,5
100	0,29	5,98	0,29	4,4
101	1,34	8,62	0	4,89
102	0,57	9,36	0	4,76
103	0,9	3,72	0	4,81
104		6,08		3,91
106	0,48	7,66	0	4,96
107	0,37	8,13	0	7,54
108	0,85	5,63	0	4,78
109	1,15	9,11	0	4,88
110	3,07	8,75	0	2,87
111	0,46	9,44	0	4,96
112	0,27	9,64	0	5,21
113				
114	0,31	8,43	0	4,74
115	0,38	9,05	0	5,91
116	0,78	9,31	0	4,89
117		7,44	0	4,9
118	1,31	9,44	0	5,08
119	0,71	9,93	0	6
120	0,54	7,2	0	5,18
121	1,39	9,94	0	5

122	1,21	8,72	0	4,94
123	0,53	9,48	0	4,96
